# Synthesis, Antifungal and Antitumor Activity of Novel (*Z*)-5-Hetarylmethylidene-1,3-thiazol-4-ones and (*Z*)-5-Ethylidene-1,3-thiazol-4-ones

**DOI:** 10.3390/molecules18055482

**Published:** 2013-05-13

**Authors:** Alberto Insuasty, Juan Ramírez, Marcela Raimondi, Carlos Echeverry, Jairo Quiroga, Rodrigo Abonia, Manuel Nogueras, Justo Cobo, María Victoria Rodríguez, Susana A. Zacchino, Braulio Insuasty

**Affiliations:** 1 Heterocyclic Compounds Research Group, Department of Chemistry, Universidad del Valle, A.A. 25360, Cali, Colombia; 2 Pharmacognosy, Faculty of Biochemical and Pharmaceutical Sciences, Universidad Nacional de Rosario, Suipacha 531, (2000), Rosario, Argentina; 3 Department of Inorganic and Organic Chemistry, Universidad de Jaén, 23071, Jaén, Spain

**Keywords:** antifungal activity, antitumor activity, hetarylmethylidenerhodanine derivatives, piperidine, morpholine

## Abstract

New hetaryl- and alkylidenerhodanine derivatives **3a**–**d**, **3e**, and **4a**–**d** were prepared from heterocyclic aldehydes **1a**–**d** or acetaldehyde **1e**. The treatment of several rhodanine derivatives **3a**–**d** and **3e** with piperidine or morpholine in THF under reflux, afforded (*Z*)-5-(hetarylmethylidene)-2-(piperidin-1-yl)thiazol-4(5*H*)-ones and 2-morpholinothiazol-4(5*H*)-ones **5a**–**d**, **6a**–**d**, and (*Z*)-5-ethylidene-2-morpholinothiazol-4(5*H*)-one (**5e**), respectively, in good yields. Structures of all compounds were determined by IR, 1D and 2D NMR and mass spectrometry. Several of these compounds were screened by the U.S. National Cancer Institute (NCI) to assess their antitumor activity against 60 different human tumor cell lines. Compound **3c** showed high activity against HOP-92 (Non-Small Cell Lung Cancer), which was the most sensitive cell line, with GI_50_ = 0.62 μM and LC_50_ > 100 μM from the *in vitro* assays. *In vitro* antifungal activity of these compounds was also determined against 10 fungal strains. Compound **3e** showed activity against all fungal strains tested, but showed high activity against *Saccharomyces cerevisiae* (MIC 3.9 μg/mL)*.*

## 1. Introduction

In recent years, the synthesis and pharmacological properties of several rhodanine derivatives have been reported [1,2]. Among them, the literature highlights the antibacterial activity of 5-arylidene rhodanine derivatives [3], antimicrobial activity of 5-hetarylidene rhodanine derivatives [4], and antifungal activity of 5-arylidene rhodanine-3-acetic acid [5] and 5-arylidene rhodanines [6]. The substitution of rhodanine derivatives at C-2 (C=S) of the ring has produced compounds with important biological activity [7]. This type of compounds has been used as precursors for the synthesis of new fused heterocyclic systems [8]. Recently, new hetarylmethylidene derivatives were synthesized by Xu and co-workers [9] from the reaction of 1,3-diarylpyrazole-4-carbaldehyde with rhodanine-3-acetic acid. These compounds showed important antimicrobial activity. Herein, we report the synthesis of some new hetarylmethylidene rhodanine derivatives and their antitumor and antifungal activities.

## 2. Results and Discussion

### 2.1. Chemistry

**Scheme 1:**
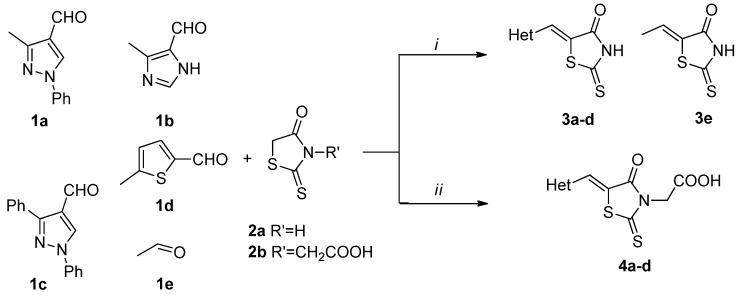
General methodology for the synthesis of rhodanine and rhodanine-3-acetic acid derivatives and their structures.

New rhodanine derivatives were prepared from heterocyclic aldehydes **1a**–**d** by different pathways, leading to the hetarylmethylidenerhodanine **3a**–**d** and the rhodanine-3-acetic acid derivatives **4a**–**d**. To obtain the expected compounds **3a**–**d**, a mixture of rhodanine **2a** with the respective heterocyclic aldehyde **1a**–**d** and catalytic amounts of piperdine was heated for 4 h at reflux in absolute ethanol. In the case of **3a**, a yellow solid was obtained which after spectroscopic characterization (IR, ^1^H and ^13^C-NMR and mass spectrometry) was confirmed to be the proposed compound. It was obtained in 86% yield (Scheme 1).

*Reagents and Conditions: i *= (R' = H), piperidine (catalytic amounts), reflux, 4 h; *ii* = (R' = CH_2_COOH), MW (100 W, 100 °C, 30 psi), 5 min.

Compound **3a** exhibited characteristic signals of its functional groups. The IR spectrum showed absorption bands at 3,134, 1,684 and 1,213 cm^−^^1^ associated with the –NH, C=O and C=S functionalities, respectively. In the ^1^H-NMR spectrum, a broad singlet at δ = 13.71 ppm was assigned to the –NH group and singlets at 7.39 and 2.40 ppm were assigned to the vinylidenic proton and to the methyl group of the pyrazole ring, respectively. The ^13^C-NMR spectrum showed signals at δ = 169.4 and 195.5 ppm assigned to the (C=O) and (C=S) functionalities, respectively. All signals agree with the proposed structure **3a**. Finally, the mass spectrum, showed a peak (*m/z* 301) corresponding to the molecular ion. Similar results were observed for compounds **3b**–**d**, obtained in good yields, as shown in Table 1.

**Table 1:** Melting points and yields for the hetarylmethylidene rhodanine derivatives **3a**–**d**, **3e** and rhodanin-3-acetic acid derivatives **4a**–**d**.

Compound	m.p. (°C)	Yield (%)
**3a**	294–295	86
**3b**	307–309	91
**3c**	315–317	86
**3d**	230–231	85
**3e**	145–147	64
**4a**	279–281	81
**4b**	254–256	53
**4c**	263–265	92
**4d**	232–234	63

Chen and co-workers have previously reported the synthesis of rhodanine-3-acetic acid derivatives in acetic acid under reflux and using sodium acetate as catalyst [10]. Here we propose the use of microwave irradiation for the synthesis of these compounds with shorter reaction times and easier works-up.

In this sense, a mixture of heterocyclic aldehyde **1a** and rhodanine-3-acetic acid was subjected to microwave irradiation (CEM-focused microwave reactor) using DMF as solvent at 100 °C and 100 W of power for 5 min, leading to the formation of a yellow solid which was characterized by IR, ^1^H and ^13^C**-**NMR and mass spectrometry to correspond to the desired compound **4a**. It was obtained in 92% yield.

In the ^1^H-NMR spectrum, we observed a broad singlet at 13.45 ppm assigned to the acid proton (–COOH) and a signal at 4.73 ppm assigned to methylene protons between the acid group and thiazole ring, while the remaining signals corresponded to rest of compound **4a**. In the ^13^C-NMR spectrum, a signal at 167.2 ppm corresponding to a carbonyl carbon (–COOH) was observed. With the help of DEPT-135 at 45.0 ppm the signal assigned to the methylene carbon between the –COOH group and the rhodanine ring was discerned. 

The same procedure was followed to obtain compounds **4b**–**d** in good yields (Scheme 1, Table 1), which highlights the efficiency of the microwave radiation for the synthesis of these compounds. The *Z*- configuration of compounds **3a**–**d** and **4a**–**d** was deduced based on the previously reported crystal structure of compounds of the (*Z*)-5-arylidenerhodanine type [11,12]. 

**Scheme 2:**
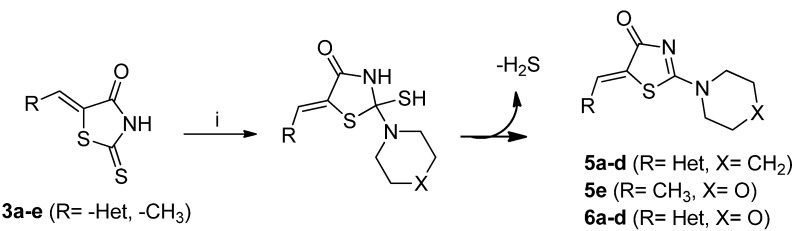
General methodology for the synthesis of (*Z*)-5-(hetarylmethylidene)-2-(piperidin-1-yl)thiazol-4(5*H*)-ones and (*Z*)-5-(hetarylmethylidene)-2-morpholinothiazol-4(5*H*)-ones and their structures.

Subsequently, compound **3a** upon reflux during 18 h with an excess of piperidine (2 equiv.) in THF afforded a white solid accompanied by the loss of H_2_S, as detected by its characteristic smell (Scheme 2). This solid corresponded to (*Z*)-5-(hetarylmethylidene)-2-(piperidin-1-yl)thiazol-4(5*H*)-one (**5a**, 85% yield), as confirmed by its IR, ^1^H, ^13^C-NMR and mass spectra. In the ^1^H-NMR spectrum of compound **5a**, a singlet at 8.1 ppm corresponding to the proton of the pyrazole ring, a singlet at 7.66 ppm corresponding to the vinylidenic proton, and two broad singlets (2H each one) at 4.03 and 3.60 ppm, assignable to the adjacent methylenes to nitrogen of the piperidine ring, were observed.

*Reagents and Conditions: i* = (R' = H), piperidine (2 equiv.), THF at reflux, 7–24 h.

In the ^13^C-NMR spectrum, the disappearance of the characteristic signal of the (C=S) carbon atom, along with the appearance of aliphatic signals at 50.3, 49.6, 26.1, 25.4 and 24.0 ppm (corresponding to the piperidine moiety), confirmed the structure proposed for compound **5a**. The mass spectrum showed a peak with (*m/z* 352) which is in accordance with the expected molecular ion for a structure like **5a**. The same procedure was followed for hetarylmethylidenic derivatives **3b**–**d**, with similar results, affording compounds **5b**–**d**, as shown in Table 2. Based on these results; we decided to extend the same methodology to the hetarylmethylidenic derivatives **3a**–**d** but using morpholine instead of piperidine. This approach led to the synthesis of the (*Z*)-5-(hetarylmethylidene)-2-morpholinothiazol 4(5*H*)-ones **6a**–**d**, Table 2.

**Table 2:** Melting points and yields for the piperidine and morpholine derivatives **5a**–**d**, **5e** and **6a**–**d**.

Compound	-X-	m.p. (°C)	Yield (%)
**5a**	-CH_2_-	141–143	85
**5b**	-CH_2_-	261–262	70
**5c**	-CH_2_-	262–264	95
**5d**	-CH_2_-	194–196	93
**5e**	-O-	193–195	45
**6a**	-O-	264–265	71
**6b**	-O-	270–272	62
**6c**	-O-	266–268	86
**6d**	-O-	206–208	85

In a further experiment, the synthesis of the (*Z*)-5-ethylidene-2-thioxothiazolidin-4-one (**3e**) was achieved by refluxing during 7 h an ethanolic solution of rhodanine, paraldehyde and catalytic amounts of piperidine. A yellow solid was obtained in 64% yield. This compound was subjected to reaction with morpholine as described above for compounds **6a**–**d**, thereby obtaining a brown solid in 45% yield, which, by IR, ^1^H and ^13^C-NMR and MS methods was characterized as the compound **5e** (Table 2).

### 2.2. *In Vitro* Antifungal Activity

Minimum Inhibitory Concentration (MIC) of compounds **3a**–**e**, **4a**–**d**, **5a**–**e** and **6a**–**d** were determined with the microbroth dilution methods M27-A3 and M38-A2 of CLSI [13,14] against a panel of 10 fungal species comprising four yeasts (*Candida albicans*, *C. tropicalis*, *Cryptococcus neoformans* and *Saccharomyces cerevisiae*), three *Aspergillus* spp. (*A. niger*, *A. fumigatus* and *A. flavus*) and three dermatophytes (*Trichophyton rubrum*, *T. mentagrophytes* and *Microsporum gypseum*]. Compounds with MICs > 250 μg/mL were considered inactive. MICs between 250–125 μg/mL were indicative of low activity; between 62.5–31.25 μg/mL, moderate activity; MICs ≤ 15.6 μg/mL, high activity. Among the last ones, compounds displaying MICs ≤ 10 μg/mL were considered of great interest for further development. In addition to MIC, active compounds (MICs ≤ 250 μg/mL) were tested for its capacity of killing fungi rather than inhibiting them through the determination of the Minimum Fungicidal Concentration (MFC). It was determined by plating an aliquote from each clear well of MIC determinations, onto a plate containing clear culture medium. After incubation, MFCs were determined as the lowest concentration of each compound showing no growth, which clearly indicated that fungi were dead rather than inhibited (the detailed methodology is explained in the Experimental section). 

Compounds **3a**, **3b**, **3d**, **4b**–**d, 5a**–**e** and **6a**–**d**, were inactive (Table 3). In contrast, compounds **3c**, **3e** and **4a** showed varied activities, being **3e** the one with the broadest and highest activity. An analysis of correlation between structure and activity showed that the most potent compound **3e** possessed the simplest structure among the all tested compounds, possessing a thiazolidine ring and methyl substituent as the R moiety. The other structure with R = methyl (compound **5e**) with a more complex structure containing a thiazole ring and morpholino substituent, did not possess activity up to 250 µg/mL.

**Table 3:** *In vitro *antifungal activities (MIC and MFC values in μg/mL, showed as MIC/MFC) of hetarylidenerhodanine derivatives.

Compound	Structure	Antifungal Activity MIC/MFC (μg/mL)									
		Ca	Ct	Sc	Cn	Afu	Afl	Ani	Mg	Tr	Tm
3a	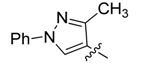	>250	>250	>250	>250	>250	>250	>250	>250	>250	>250
3b	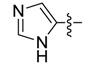	>250	>250	>250	>250	>250	>250	>250	>250	>250	>250
3c	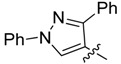	>250	>250	>250	125/125	250/250	250/250	250/250	125/125	125/125	125/125
3d	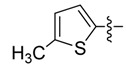	>250	>250	>250	>250	>250	>250	>250	<250	<250	<250
3e	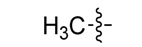	7.8/31.2	7.8/31.2	3.9/15.6	15.6/62.5	31.2/250	31.2/250	62.5/250	7.8/7.8	7.8/15.6	15.6/15.6
4a	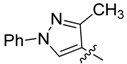	>250	>250	>250	>250	>250	>250	>250	125/125	62.5/62.5	62.5/62.5
4b	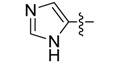	>250	>250	>250	>250	>250	>250	>250	>250	>250	>250
4c	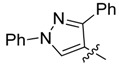	>250	>250	>250	>250	>250	>250	>250	>250	>250	>250
4d	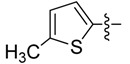	>250	>250	>250	>250	>250	>250	>250	>250	>250	>250
5a	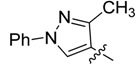	>250	>250	>250	>250	>250	>250	>250	>250	>250	>250
5b	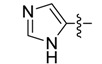	>250	>250	>250	>250	>250	>250	>250	>250	>250	>250
5c	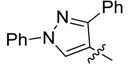	>250	>250	>250	>250	>250	>250	>250	>250	>250	>250
5d	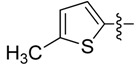	>250	>250	>250	>250	>250	>250	>250	>250	>250	>250
5e	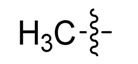	>250	>250	>250	>250	>250	>250	>250	>250	>250	>250
6a	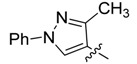	>250	>250	>250	>250	>250	>250	>250	>250	>250	>250
6b	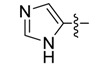	>250	>250	>250	>250	>250	>250	>250	>250	>250	>250
6c	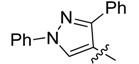	>250	>250	>250	>250	>250	>250	>250	>250	>250	>250
6d	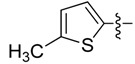	>250	>250	>250	>250	>250	>250	>250	>250	>250	>250
amphotericin B	-	0.78		0.50	0.25	0.50	0.50	0.50	0.12	0.07	0.07
ketoconazole	-	1.56		3.12	0.39	0.78	0.78	1.56	0.04	0.01	0.02
terbinafine	-	0.50		0.50	0.25	0.12	0.50	0.25	0.05	0.02	0.02

Antifungal activity was determined with the microbroth dilution assay following the CLSI guidelines. Fungi used: *C.a.*: *Candida albicans* ATCC10231, *C.t.*: *Candida tropicalis* C131; *C.n.*: *Cryptococcus neoformans* ATCC32264, *S.c.*: *Saccharomyces cerevisiae* ATCC9763, *A.n.*: *Aspergillus niger* ATCC9029, *A.fl.*: *Aspergillus flavus* ATCC 9170, *A.fu.*: *Aspergillus fumigatus* ATCC 26934, *M.g.*: *Microsporum gypseum* C 115, *T.r.*: *Trichophyton rubrum* C113, *T.m.*: *Trichophyton mentagrophytes* ATCC 9972.

An analysis of the effect of the substituents other than the methyl group showed that those with 4-methyl-1*H*-imidazol-5-yl [compounds named “b” (**3**, **4**, **5** and **6**)] or with 5-methylthiophen-2-yl [named “**d**” (**3**, **4**, **5** and **6**)] were inactive. On the other hand, among compounds with R = 1,3-diphenyl-1*H*-pyrazol-4-yl (named “**c**”), or R = 1-methyl-3-phenyl-1*H*-pyrazol-4-yl (named “**a**”), **3c** showed low broad spectrum of activity and **4a** displayed moderate activity (MICs between 62.5–125 μg/mL) respectively. Interestingly, the three active structures (**3c**, **3e** and **4a**) possess fungicidal rather than fungistatic activities, with MFC values between 7.8 and 250 µg/mL. Compound **3e** showed the lowest MFC values against *S. cerevisiae* and the dermatophytes *M. gypseum*, *T. rubrum* and *T. mentagrophytes*.

### 2.3. *In Vitro* Antitumor Activity 

All compounds synthesized were sent to the U.S. National Cancer Institute (NCI) to evaluate antitumor activity. The results showed that only compound **3c** had an interesting antitumor activity and therefore was evaluated against 60 different cell lines (melanoma, leukemia, lung cancer, colon, brain, breast, ovary, kidney and prostate). In order to determine its cytostatic activity compound **3c** was evaluated at five concentrations (100, 10, 1.0, 0.1 and 0.001 μM). Compound **3c** shows an interesting activity against CCRF-CEM and RPMI-8226 (leukemia) (GI_50_: 2.50, 2.52 μM and LC_50_ >100 μM) respectively.

It also exhibited activity against EKVX and NCI-H522 (Non-Small Cell Lung Cancer) (GI_50_: 3.03, 2.96 μM and LC_50_ >100 μM), the most sensitive cell line was HOP-92 (Non-Small Cell Lung Cancer) (GI_50_: 0.62 μM and LC_50_ >100 μM). These results although moderate, open the research on these compounds with the aim of finding new potential antitumor agents. The LC_50_ found indicates a low toxicity of such compounds for normal human cell lines, as required for potential anti-tumor agents (see Table 4).

**Table 4:** *In vitro* testing expressed as growth inhibition of cancer cell lines for compound **3c** ^a^.

Panel/Cell Line	Compound 3c	
	GI_50_^b^ (μM)	LC_50_^c^ (μM)
*Leukemia*		
CCRF-CEM	2.50	>100
HL-60(TB)	4.83	>100
K562	7.54	>100
MOLT-4	14.8	>100
RPMI-8226	2.52	>100
SR	7.29	>100
*Non Small Cell Lung*		
A549/ATCC	5.88	>100
EKVX	3.03	>100
HOP-62	22.7	>100
HOP-92	0.62	>100
NCI-H226	2.03	>100
NCI-H23	2.68	>100
NCI-H322M	7.63	>100
NCI-H460	5.50	54.4
NCI-H522	2.96	>100
*Colon Cancer*		
COLO 205	21.2	>100
HCC-2998	6.05	>100
HCT-116	5.62	70.2
HCT-15	4.71	96.2
HT29	12.5	>100
KM12	6.24	63.5
SW-620	19.6	>100
*Prostate Cancer*		
PC-3	5.66	>100
DU-145	12.6	>100
*CNS Cancer*		
SF-268	17.2	>100
SF-295	3.33	82.6
SF-539	5.53	61.0
SNB-19	6.14	>100
SNB-75	17.8	>100
U251	5.54	64.8
*Melanoma*		
LOX IMVI	10.0	>100
MALME-3M	3.84	6.11
M14 0.405	6.75	>100
MDA-MB-435	4.91	>100
SK-MEL-2	4.18	70.7
SK-MEL-28	9.22	>100
SK-MEL-5	3.19	58.4
UACC-257	13.2	>100
UACC-62	3.36	5.77
*Renal Cancer*		
786-0	3.92	>100
A498	2.99	94.4
ACHN	7.40	52.1
CAKI-1	7.15	>100
RXF 393	22.4	>100
SN12C	9.93	>100
TK-10	8.00	>100
UO-31	4.39	>100
*Breast Cancer*		
MCF7	8.31	>100
MDA-MB231/ATCC	10.1	>100
HS 578T	6.03	>100
BT-549	5.20	86.5
T-47D	4.59	>100
MDA-MB-468	6.83	>100

^a^ Data obtained from NCI’s *in vitro* disease-oriented human tumor cell lines screen [15]; ^b^ GI_50_ was the drug concentration resulting in a 50% reduction in the net protein increase (as measured by SRB staining) compared to control cells during the drug incubation; Determined at five concentration levels (100, 10, 1.0, 0.1 and 0.01 mM); ^c^ LC_50_ is a parameter of cytotoxicity and reflects the molar concentration needed to kill 50% of the cells.

## 3. Experimental

### 3.1. General

Reagents and solvents used below were obtained from commercial sources. Melting points were measured using a Stuart SMP3 melting point device. IR spectra were obtained with a Shimadzu IRAffinity-1. The ^1^H and ^13^C-NMR spectra were run on a Bruker DPX 400 spectrometer operating at 400 and 100 MHz respectively, using DMSO-*d*_6_ and CDCl_3_ as solvents and TMS as internal standard. The mass spectrum was obtained on a Shimadzu-GCMS-QP2010 spectrometer operating at 70 eV. Microwave experiments were carried out on a focused microwave reactor (300W CEM Discover) Thin layer chromatography (TLC) was performed on a 0.2-mm pre-coated plates of silica gel 60GF254 (Merck, Darmstadt, Germany).

### 3.2. Synthesis

#### 3.2.1. General Procedure for the Synthesis of (*Z*)-5*-*Hetarylmethylidene-2-thioxothiazolidin-4-ones **3a**–**d**

Two drops of piperidine (0.01 equiv.) were added to an ethanolic solution of heterocyclic aldehyde (**1a**–**d**, 1.1 mmol) and rhodanine (**2a**, 1 mmol). The mixture was refluxed for 4–6 h and the solid formed was isolated by vacuum filtration and washed with cold ethanol.

*(Z)-5-((3-Methyl-1-phenyl-1H-pyrazol-4-yl)methylidene)-2-thioxothiazolidin-4-one* (**3a**). Yellow solid (86%), m.p. 294–295 °C; FT-IR (KBr), υ: (NH) 3134, (C=O) 1684 and (C=S) 1213 cm^−^^1^; ^1^H-NMR (DMSO-*d*_6_), δ: 2.40 (s, 3H, CH_3_), 7.36 (t, *J* = 7.44 Hz, 1H, Ar-H*_p_*), 7.39 (s, 1H, H-6), 7.51 (dd, *J* = 7.44 and 8.52 Hz, 2H, Ar-H*_m_*), 7.93 (d, *J* = 8.52 Hz, 2H, Ar-H*_o_*), 8.50 (s, 1H, H-5’), 13.71 (s, 1H, -NH-) ppm; ^13^C-NMR (DMSO-*d*_6_), δ: 12.0 (-CH_3_), 117.0 (C-4'), 119.4 (C*_o_*), 122.3 (C-6), 123.4 (C-5), 127.5 (C*_p_*), 128.0 (C-5'), 130.0 (C*_m_*), 139.1 (C*_i_*), 152.4 (C-3'), 169.4 (C=O), 195.5 (C=S) ppm. MS (EI, 70 eV) *m/z* (%): 301 (M^+^, 50), 214 (100), 213 (55), 129 (22), 109 (18), 107 (13), 104 (10), 102 (16), 96 (12), 77 (71), 70 (19), 69 (12). Anal. Calcd. for C_14_H_11_N_3_OS_2_ (301.03): C, 55.79%; H, 3.68%; N, 13.94%; found: C, 56.02%; H, 3.71%; N, 13.56%.

*(Z)-5-((4-Methyl-1H-imidazol-5-yl)methylidene)-2-thioxothiazolidin-4-one* (**3b**). Orange crystalline solid (91%), m.p. 307–309 °C; FT-IR (KBr), υ: (NH) 3558, (NH) 3225, (C=O) 1690 and (C=S) 1217 cm^−^^1^; ^1^H-NMR (DMSO-*d*_6_), δ: 2.39 (s, 3H, CH_3_), 7.49 (s, 1H, H-6), 7.83 (s, 1H, H-2'), 12.61 (s, 1H, NH-1'), 13.31 (s, 1H, NH-3) ppm; ^13^C-NMR (DMSO-*d*_6_), δ: 9.7 (-CH_3_), 120.2 (C-5), 123.5 (C-6), 132.7 (C-5' or C-4'), 135.4 (C-5' or C-4'), 137.6 (C-2'), 169.7 (C=O), 200.2 (C=S) ppm. MS (IE, 70 eV) *m/z* (%): 225 (M^+^, 38), 139 (10), 138 (70), 137 (100), 69 (25), 42 (13). Anal. Calcd. for C_8_H_7_N_3_OS_2_ (225.00): C, 46.25%; H, 3.13%; N, 18.65%; found: C, 46.09%; H, 3.10%; N, 18.98%. 

*(Z)-5-((1,3-Diphenyl-1H-pyrazol-4-yl)methylidene)-2-thioxothiazolidin-4-one* (**3c**). Yellow solid (86%), m.p. 315–317 °C; FT-IR (KBr), υ: (NH) 3134, (C=O) 1695 and (C=S) 1223 cm^−^^1^; ^1^H-NMR (DMSO-*d*_6_), δ: 7.42 (t, *J* = 7.40 Hz, 1H, Ar-H*_p_*), 7.46 (s, 1H, H-6), 7.51–7.59 (m, 5H, Ar-H*_m, m',p'_*), 7.66 (d, *J* = 8.08 Hz, 2H, Ar-H*_o'_*), 7.99 (d, *J* = 8.24 Hz, 2H, Ar-H*_o_*), 8.63 (s, 1H, H-5') ppm; ^13^C-NMR (DMSO-*d*_6_), δ: 116.3 (C-4'), 120.0 (C*_o_*), 122.3 (C-6), 125.3 (C-5), 128.0 (C*_p_*) 129.0 (C-5'), 129.1 (C*_o'_*), 129.3 (C*_m'_*), 129.4 (C*_p'_*), 130.0 (C*_m_*), 139.1 (C*_i'_*), 139.4 (C*_i_*), 154.3 (C-3'), 169.3 (C=O), 195.4 (C=S) ppm. MS (IE, 70 eV) *m/z* (%): 363 (M^+^, 76), 277 (21), 276 (100), 275 (39), 243 (10), 242 (10), 215 (17), 172 (13), 77 (65), 28 (10). Anal. Calcd. for C_19_H_13_N_3_OS_2_ (363.05): C, 62.79%; H, 3.61%; N, 11.56%; found: C, 62.57%; H, 3.89%; N, 11.32%.

*(Z)-5-((5-Methylthiophen-2-yl)methylidene)-2-thioxo-thiazolidin-4-one* (**3d**). Orange solid (85%), m.p. 230–231 °C; FT-IR (KBr), υ: (NH) 3143, (C=O) 1689 and (C=S) 1199 cm^−^^1^; ^1^H-NMR (DMSO-*d*_6_), δ: 2.56 (s, 3H, CH_3_), 7.02 (d, *J* = 3.71 Hz, 1H, H-4') 7.54 (d, *J* = 3.71 Hz, 1H, H-3'), 7.82 (s, 1H, H-6), 13.71 (s, 1H, NH) ppm; ^13^C-NMR (DMSO-*d*_6_), δ: 16.1 (-CH_3_), 121.9 (C-5), 125.6 (C-6), 128.6 (C-3'), 135.9 (C-5'), 136.6 (C-4'), 149.9 (C-2'), 169.5 (C=O), 195.1 (C=S) ppm. MS (IE, 70 eV) *m/z* (%): 241 (M^+^, 34), 155 (14), 154 (100), 153 (52), 121 (21), 97 (16), 77 (18), 69 (12), 59 (13). Anal. Calcd. for C_9_H_7_NOS_3_ (240.97): C, 44.79%; H, 2.92%; N, 5.80%; found: C, 45.08%; H, 3.11%; N, 5.85%.

*(Z)-5-Ethylidene-2-thioxothiazolidin-4-one* (**3e**). Two drops of piperidine (0.01 equiv.) were added to an ethanolic solution of rhodanine (**2a**, 1 mmol) and paraldehyde (1.1 mmol). The mixture was refluxed for 7 h, the solution was cooled and crushed ice was added and the solid formed was isolated by vacuum filtration and washed with hexane and water. Purification was carried out by column chromatography silica gel using a mixture of CHCl_3_–EtOAc (30:1) as eluent. The solvent was removed under reduced pressure. This compound was obtained as a yellow solid (64%), m.p. 145–147 °C; FT-IR (KBr), υ: (NH) 3161, (C=O) 1703 and (C=S) 1219 cm^−^^1^; ^1^H-NMR (CDCl_3_), δ: 1.98 (d, *J* = 7.32 Hz, 3H, CH_3_), 6.99 (q, *J* = 7.32 Hz, 1H, H-6) 9.75 (s, 1H, NH) ppm; ^13^C-NMR (CDCl_3_), δ: 17.4 (–CH_3_), 130.4 (C-5), 134.1 (C-6), 167.2 (C=O), 193.6 (C=S) ppm. MS (IE, 70 eV) *m/z* (%): 159 (M^+^, 100), 100 (25), 72 (66), 71 (36), 45 (7). Anal. Calcd. for C_5_H_5_NOS_2_ (158.98): C, 37.72%; H, 3.17%; N, 8.80%; found: C, 37.65%; H, 3.22%; N, 8.43%.

#### 3.2.2. General Procedure for the Synthesis of 2-(5*Z*)-(Hetarylmethylidene)-4-oxo-2-thioxothiazolidin-3-yl) Acetic Acids **4a**–**d**

A mixture of heterocyclic aldehyde (**1a**–**d**, 0.4 mmol) and rhodanine-3-acetic acid (**2b**, 0.4 mmol) in DMF, was subjected to irradiation with microwaves for 5 min at 100 °C and 100 W. Then, a mixture of ethanol:water (1:1) was added and the solid formed was isolated by vacuum filtration and washed with ethanol.

*(Z)-5-((3-Methyl-1-phenyl-1H-pyrazol-4-yl)methylidene)-4-oxo-2-thioxothiazolidin-3-yl acetic acid* (**4a**). Yellow solid (81%), m.p. 279–281 °C; FT-IR (KBr), υ: (–COOH) 3315, (C=O) 1715 and (C=S) 1323 cm^−^^1^; ^1^H-NMR (DMSO-*d*_6_), δ: 2.43 (s, 3H, CH_3_), 4.73 (s, 2H, –N–CH_2_–COOH), 7.38 (t, *J* = 7.45 Hz, 1H, Ar-H*_p_*), 7.52 (dd, *J* = 7.45 and 7.95 Hz, 2H, Ar-H*_m_*), 7.61 (s, 1H, H-6), 7.95 (d, *J* = 7.95 Hz, 2H, Ar-H*_o_*), 8.61 (s, 1H, H-5'), 13.45 (br. s., 1H, –COOH) ppm; ^13^C-NMR (DMSO-*d*_6_), δ: 11.51 (–CH_3_), 45.0 (–N–CH_2_–COOH), 116.5 (C-4'), 119.0 (C*_o_*), 124.1 (C*_p_*), 127.1 (C-5), 128.1 (C-6), 129.4 (C-5'), 132.1 (C*_m_*), 138.6 (C*_i_*), 152.2 (C-3'),165.9 (–COOH), 167.2 (C=O), 192.6 (C=S) ppm. MS (IE, 70 eV) *m/z* (%): 359 (M^+^, 26), 214 (91), 129 (34), 117 (80), 72 (100). Anal. Calcd. for C_16_H_13_N_3_O_3_S_2_ (359.04): C, 53.47%; H, 3.65%; N, 11.69%; found: C, 53.77%; H, 3.89%; N, 11.77%.

*(Z)-5-((4-Methyl-1H-imidazol-5-yl)methylidene)-4-oxo-2-thioxothiazolidin-3-yl acetic acid* (**4b**). Yellow solid (53%), m.p. 254–256 °C; FT-IR (KBr), υ: (COOH) 3368, (C=O) 1718 and (C=S) 1319 cm^−^^1^; ^1^H-NMR (DMSO-*d*_6_), δ: 2.42 (s, 3H, CH_3_), 4.68 (s, 2H, –N–CH_2_–COOH), 7.69 (s, 1H, H-6), 7.86 (s, 1H, H-2'), 12.70 (s, 1H, NH), 13.71 (br. s., 1H, –COOH) ppm; ^13^C-NMR (DMSO-*d*_6_), δ: 9.3 (–CH_3_), 44.8 (–N–CH_2_–COOH), 116.1 (C-5), 124.7 (C-6), 132.4 (C-5'), 135.8 (C-4'), 137.3 (C-2'), 166.3 (–COOH), 167.6 (C=O), 197.4 (C=S) ppm. MS (IE, 70 eV) *m/z* (%): 283 (M^+^, 51), 166 (9), 139 (13), 138 (100), 72 (13). Anal. Calcd. for C_10_H_9_N_3_O_3_S_2_ (283.01): C, 42.39%; H, 3.20%; N, 14.83%; found: C, 42.55%; H, 4.10%; N, 14.44%.

*(Z)-5-((1,3-Diphenyl-1H-pyrazol-4-yl)methylidene)-4-oxo-2-thioxothiazolidin-3-yl acetic acid* (**4c**). Yellow solid (92%), m.p. 263–265 °C; FT-IR (KBr), υ: (COOH) 3320, (C=O) 1715 and (C=S) 1320 cm^−^^1^; ^1^H-NMR (DMSO-*d*_6_), δ: 4.67 (s, 2H, –N–CH_2_–COOH), 7.42 (t, *J* = 8.00 Hz, 1H, Ar-H*_p_*), 7.54–7.58 (m, 5H, Ar-H*_m, m',p'_*), 7.64 (d, *J* = 7.90 Hz, 2H, Ar-H*_o'_*), 7.66 (s, 1H, H-6), 8.04 (d, *J* = 8.03 Hz, 2H, Ar-H*_o_*), 8.80 (s, 1H, H-5') 13.23 (br. s., 1H, –COOH) ppm; ^13^C-NMR (DMSO-*d*_6_), δ: 44.79 (–N–CH_2_–COOH), 115.4 (C-4'), 119.4 (C*_o_*), 120.7 (C-6), 123.8 (C-5), 127.6 (C*_o’_*), 128.7 (C-5'), 128.9 (C*_p_*), 129.1 (C*_m'_*), 129.5.0 (C*_p'_*), 131.0 (C*_m_*), 138.6 (C*_i'_*), 139.6 (C*_i_*), 154.0 (C-3'), 166.0 (–COOH), 167.2 (C=O), 192.6 (C=S) ppm. MS (IE, 70 eV) *m/z* (%): 421 (M^+^, 30), 276 (100), 72 (54). Anal. Calcd. for C_21_H_15_N_3_O_3_S_2_ (421.06): C, 59.84%; H, 3.59%; N, 9.97%; found: C, 60.14%; H, 3.67%; N, 10.12%.

*(5Z)-((5-Methylthiophen-2-yl)methylidene)-4-oxo-2-thioxothiazolidin-3-yl acetic acid* (**4d**). Orange solid (63%), m.p. 232–234 °C; FT-IR (KBr), υ: (COOH) 3320, (C=O) 1712 and (C=S) 1321 cm^−^^1^; ^1^H-NMR (DMSO-*d*_6_), δ: 2.53 (s, 3H, CH_3_), 4.63 (s, 2H, –N–CH_2_–COOH), 7.05 (d, *J* = 3.70 Hz, 1H, H-4') 7.61 (d, *J* = 3.70 Hz, 1H, H-3'), 8.04 (s, 1H, H-6), 13.42 (br. s., 1H, –COOH) ppm; ^13^C-NMR (DMSO-*d*_6_), δ: 16.0 (–CH_3_), 45.82 (–N–CH_2_–COOH), 122.1 (C-5), 126.8 (C-6), 128.3 (C-3'), 136.9 (C-5'), 138.2 (C-4'), 147.1 (C-2'), 165.5 (–COOH), 167.9 (C=O), 191.8 (C=S) ppm. MS (IE, 70 eV) *m/z* (%): 299 (M^+^, 31), 154 (100), 121 (15), 7 (8), 45 (10). Anal. Calcd. for C_11_H_9_NO_3_S_3_ (421.06): C, 44.13%; H, 3.03%; N, 4.68%; found: C, 44.19%; H, 3.15%; N, 4.72%.

#### 3.2.3. General Procedure for the Synthesis of 5*-*Hetarylmethylidene-2-(piperidin-1-yl)thiazol-4-ones, 5-hetarylmethylidene-2-morpholinothiazol-4-ones and (*Z*)-5-Ethylidene-2-morpholinothiazol-4(5*H*)-ones **5a**–**d, 5e** and **6a**–**d**

A mixture of piperidine or morpholine (2 mmol) and hetarylidene rhodanine derivatives **3a**–**b** (1 mmol) or ethylene derivative **3e** was refluxed in THF for 7–24 h. Crushed ice was added and the solid formed was isolated by vacuum filtration and washed with water and hexane.

*(Z)-5-((3-Methyl-1-phenyl-1H-pyrazol-4-yl)methylidene)-2-(piperidin-1-yl)thiazol-4(5H)-one* (**5a**). White solid (85%), m.p. 141–143 °C; FT-IR (KBr), υ: (C=O) 1680 and (C=N, C=C) 1615, 1575, 1547 cm^−^^1^; ^1^H-NMR (CDCl_3_), δ: 1.79 (br. s, 6H, –CH_2_–CH_2_–CH_2_–), 2.47 (s, 3H, CH_3_), 3.60 (br. s, 2H, N-CH_2_), 4.03 (br. s, 2H, N-CH_2_), 7.33 (t, *J* = 7.48 Hz, 1H, Ar-H*_p_*), 7.49 (dd, *J* = 7.48 and 8.51 Hz, 2H, Ar-H*_m_*), 7.66 (s, 1H, H-6), 7.69 (d, *J* = 8.51 Hz, 2H, Ar-H*_o_*), 8.01 (s, 1H, H-5') ppm; ^13^C-NMR (CDCl_3_), δ: 12.0 (–CH_3_), 24.1 (–CH_2_–CH_2_–CH_2_–), 49.6 (N-CH_2_), 50.3 (N-CH_2_), 118.0 (C-4'), 119.2 (C*_o_*), 120.8 (C-6), 126.1 (C-5), 126.5 (C-5'), 126.9 (C*_p_*), 129.5 (C*_m_*), 139.5 (C*_i_*), 152.0 (C-3'), 173.0 (C-2) 180.8 (C=O) ppm. MS (IE, 70 eV) *m/z* (%): 352 (M^+^, 20), 242 (7), 215 (15), 214 (100), 213 (29), 129 (6), 77 (8). Anal. Calcd. for C_19_H_20_N_4_OS (352.14): C, 64.75%; H, 5.72%; N, 15.90%; found: C, 64.42%; H, 5.88%; N, 15.63%.

*(Z)-5-((4-Methyl-1H-imidazol-5-yl)methylidene)-2-(piperidin-1-yl)thiazol-4(5H)-one* (**5b**). Yellow solid (70%), m.p. 261–262 °C; FT-IR (KBr), υ: (NH) 3294, (C=O) 1663 and (C=N, C=C) 1609, 1557, 1539 cm^−^^1^; ^1^H-NMR (CDCl_3_), δ: 1.74 (br. s, 6H, –CH_2_–CH_2_–CH_2_–), 2.42 (s, 3H, CH_3_), 3.62 (br. s, 2H, N-CH_2_), 3.98 (br. s, 2H, N-CH_2_), 7.66 (s, 1H, H-6), 7.72 (s, 1H, H-2'), 10.81 (s, 1H, NH) ppm; ^13^C-NMR (CDCl_3_), δ: 9.7 (–CH_3_), 26.1 (–CH_2_–CH_2_–CH_2_–), 49.2 (N-CH_2_), 50.1 (N-CH_2_), 121.5 (C-6), 124.3 (C-5), 131.4 (C-5' o C-4'), 133.0 (C-5' o C-4'), 135.5 (C-2'), 177.1 (C-2), 182.3 (C=O) ppm. MS (IE, 70 eV) *m/z* (%): 276 (M^+^, 48), 166 (10), 139 (12), 138 (100), 137 (25). Anal. Calcd. for C_13_H_16_N_4_OS (276.10): C, 56.50%; H, 5.84%; N, 20.27%; found: C, 56.32%; H, 5.51%; N, 20.32%.

*(Z)-5-((1,3-Diphenyl-1H-pyrazol-4-yl)methylidene)-2-(piperidin-1-yl)thiazol-4(5H)-one* (**5c**). Yellow solid (95%), m.p. 262–264 °C; FT-IR (KBr), υ: (C=O) 1695 and (C=N, C=C) 1606, 1572, 1535 cm^−^^1^; ^1^H-NMR (CDCl_3_), δ: 1.84 (br. s, 6H, –CH_2_–CH_2_–CH_2_–), 3.61 (br. s, 2H, N-CH_2_), 4.03 (br. s, 2H, N-CH_2_), δ 7.38 (t, *J* = 7.38 Hz, 1H, Ar-H*_p_*), 7.43–7.55 (m, 5H, Ar-H*_m, m',p'_*), 7.70 (d, *J* = 8.20 Hz, 2H, Ar-H*_o'_*), 7.82 (s, 1H, H-6), 7.83 (d, *J* = 8.64 Hz, 2H, Ar-H*_o_*), 8.20 (s, 1H, H-5') ppm; ^13^C-NMR (CDCl_3_), δ: 24.0 (–CH_2_–CH_2_–CH_2_–), 25.4 (–CH_2_–CH_2_–CH_2_–), 26.2 (–CH_2_–CH_2_–CH_2_–), 49.6 (N-CH_2_), 50.3 (N-CH_2_), 117.5 (C-4'), 119.5 (C*_o_*), 121.6 (C-6), 126.8 (C-5'), 127.3 (C*_p_*) 127.8 (C-5), 128.7 (C*_p'_*), 128.8 (C*_m'_*), 128.9 (C*_o'_*), 129.6 (C*_m_*), 131.8 (C*_i'_*), 139.5 (C*_i_*), 154.5 (C-3'), 173.0 (C-2), 180.5 (C=O) ppm. MS (IE, 70 eV) *m/z* (%): 414 (M^+^, 6), 276 (17), 109 (15), 38 (37), 36 (100), 18 (10), 17 (22), 16 (15). Anal. Calcd. for C_24_H_22_N_4_OS (414.15): C, 69.54%; H, 5.35%; N, 13.52%; found: C, 69.79%; H, 5.39%; N, 14.02%.

*(Z)-5-((5-Methylthiophen-2-yl)methylidene)-2-(piperidin-1-yl)thiazol-4(5H)-one* (**5d**). Orange solid (93%), m.p. 194–196 °C; FT-IR (KBr), υ: (C=O) 1662 and (C=N, C=C) 1600, 1574 cm^−^^1^; ^1^H-NMR (CDCl_3_), δ: 1.72 (br. s, 6H, –CH_2_–CH_2_–CH_2_–), 2.56 (s, 3H, CH_3_), 3.59 (br. s, 2H, N-CH_2_), 4.01 (br. s, 2H, N-CH_2_), 6.82 (d, *J* = 3.40 Hz, 1H, H-4') 7.15 (d, *J* = 3.40 Hz, 1H, H-3'), 7.87 (s, 1H, H-6) ppm; ^13^C-NMR (CDCl_3_), δ: 15.8 (–CH_3_), 24.1 (–CH_2_–CH_2_–CH_2_–), 25.4 (–CH_2_–CH_2_–CH_2_–), 26.2 (–CH_2_–CH_2_–CH_2_–), 49.6 (N-CH_2_), 50.3 (N-CH_2_), 124.3 (C-6), 125.5 (C-5), 127.0 (C-4'), 132.5 (C-3'), 137.3 (C-2'), 145.6 (C-5'), 173.8 (C-2), 180.9 (C=O) ppm. MS (IE, 70 eV) *m/z* (%): 292 (M^+^, 24), 155 (12), 154 (100), 153 (23). Anal. Calcd. for C_14_H_16_N_2_OS_2_ (292.07): C, 57.50%; H, 5.52%; N, 9.58%; found: C, 57.62%; H, 5.68%; N, 9.71%.

*(Z)-5-Ethylidene-2-morpholinothiazol-4(5H)-one* (**5e**). Brown solid (45%), m.p. 193–195 °C; FT-IR (KBr), υ: (C=O) 1699 and (C=N, C=C) 1639, 1554 cm^−^^1^; ^1^H-NMR (CDCl_3_), δ: 2.00 (d, *J* = 7.12 Hz, 3H, CH_3_), 3.56 (br. s, 2H, N-CH_2_), 3.81 (br. s, 4H, –CH_2_–O–CH_2_–), 4.05 (br. s, 2H, N-CH_2_), 6.99 (q, *J* = 7.12 Hz, 1H, H-6) ppm; ^13^C-NMR (CDCl_3_), δ: 18.3 (–CH_3_), 48.3 (N-CH_2_), 48.7 (N-CH_2_), 66.2 (–OCH_2_–), 66.3 (–OCH_2_–), 130.8 (C-6), 133.1 (C-5), 173.0 (C-2) 175.0 (C=O). ppm. MS (IE, 70 eV) *m/z* (%): 212 (M^+^, 100), 184 (9), 113 (27), 72 (86), 71 (23), 69 (12), 42 (11). Anal. Calcd. for C_9_H_12_N_2_O_2_S (212.06): C, 50.92%; H, 5.70%; N, 13.20%; found: C, 50.15%; H, 5.87%; N, 13.02%.

*(Z)-5-((3-Methyl-1-phenyl-1H-pyrazol-4-yl)methylidene)-2-morpholinothiazol-4(5H)-one* (**6a**). White solid (71%), m.p. 264–265 °C; FT-IR (KBr), υ: (C=O) 1683 and (C=N, C=C) 1616, 1559, 1502 cm^−^^1^; ^1^H-NMR (CDCl_3_), δ: 2.47 (s, 3H, CH_3_), 3.66 (br. s, 2H, N-CH_2_), 3.85 (br. s, 4H, –CH_2_–O–CH_2_–), 4.10 (br. s, 2H, N-CH_2_), 7.32 (t, *J* = 7.46 Hz, 1H, Ar-H*_p_*), 7.47 (dd, *J* = 7.46 and 8.07 Hz, 2H, Ar-H*_m_*), 7.68 (s, 1H, H-6), 7.69 (d, *J* = 8.07 Hz, 2H, Ar-H*_o_*), 8.01 (s, 1H, H-5') ppm; ^13^C-NMR (CDCl_3_), δ: 11.9 (–CH_3_), 48.6 (N-CH_2_), 48.8 (N-CH_2_), 66.2 (–OCH_2_–), 66.3 (–OCH_2_–), 117.8 (C-4'), 119.2 (C*_o_*), 121.8 (C-6), 125.6 (C-5), 126.1 (C-5'), 127.1 (C*_p_*), 129.5 (C*_m_*), 139.4 (C*_i_*), 152.1 (C-3'), 174.0 (C-2) 180.3 (C=O) ppm. MS (IE, 70 eV) *m/z* (%): 354 (M^+^, 34), 215 (16), 214 (100), 213 (37), 129 (12), 109 (13), 77 (29), 18 (16). Anal. Calcd. for C_18_H_18_N_4_O_2_S (354.12): C, 61.00%; H, 5.12%; N, 15.81%; found: C, 60.73%; H, 5.32%; N, 15.42%. 

*(Z)-5-((4-Methyl-1H-imidazol-5-yl)methylidene)-2-morpholinothiazol-4(5H)-one* (**6b**). Yellow solid (62%), m.p. 270–272 °C; FT-IR (KBr), υ: (NH) 3389, (C=O) 1664 and (C=N, C=C) 1605, 1540 cm^−^^1^; ^1^H-NMR (CDCl_3_), δ: 2.44 (s, 3H, CH_3_), 3.68 (br. s, 2H, N-CH_2_), 3.81 (br. s, 4H, –CH_2_–O–CH_2_–), 4.06 (br. s, 2H, N-CH_2_), 7.70 (s, 1H, H-2'), 7.71 (s, 1H, H-6), 10.23 (s, 1H, NH) ppm; ^13^C-NMR (CDCl_3_), δ: 9.6 (–CH_3_), 48.2 (N-CH_2_), 48.5 (N-CH_2_), 66.3 (–OCH_2_–), 121.9 (C-6), 124.5 (C-5), 130.9 (C-5' o C-4'), 133.1 (C-5' o C-4'), 135.1 (C-2'), 178.3 (C-2), 181.6 (C=O) ppm. MS (IE, 70 eV) *m/z* (%): 278 (M^+^, 18), 138 (26), 85 (28), 73 (33), 69 (37), 60 (52), 57 (42), 55 (36), 44 (100), 43 (99). Anal. Calcd. for C_12_H_14_N_4_O_2_S (278.08): C, 51.78%; H, 5.07%; N, 20.13%; found: C, 52.03%; H, 5.15%; N, 20.28%. 

*(Z)-5-((1,3-Diphenyl-1H-pyrazol-4-yl)methylidene)-2-morpholinothiazol-4(5H)-one* (**6c**). White solid (86%), m.p. 266–268 °C; FT-IR (KBr), υ: (C=O) 1681 and (C=N, C=C) 1602, 1567, 1502 cm^−^^1^; ^1^H-NMR (CDCl_3_), δ: 3.66 (br. s, 2H, N-CH_2_), 3.86 (br. s, 4H, –CH_2_–O–CH_2_–), 4.10 (br. s, 2H, N-CH_2_), 7.39 (t, *J* = 7.44 Hz, 1H, Ar-H*_p_*), 7.43–7.55 (m, 5H, Ar-H*_m, m',p'_*), 7.70 (d, *J* = 8.17 Hz, 2H, Ar-H*_o'_*), 7.82 (d, *J* = 8.62 Hz, 2H, Ar-H*_o_*), 7.85 (s, 1H, H-6), 8.19 (s, 1H, H-5') ppm; ^13^C-NMR (CDCl_3_), δ: 48.6 (N-CH_2_), 48.8 (N-CH_2_), 66.2 (–OCH_2_–), 66.3 (–OCH_2_), 117.3 (C-4'), 119.5 (C*_o_*), 122.6 (C-6), 126.8 (C-5'), 126.9 (C-5), 127.4 (C*_p_*), 128.8 (C*_p'_*), 128.9 (C*_m'_*), 129.0 (C*_o'_*), 129.6 (C*_m_*), 131.7 (C*_i'_*), 139.4 (C*_i_*), 154.6 (C-3'), 174.0 (C-2), 180.1 (C=O) ppm. MS (IE, 70 eV) *m/z* (%): 416 (M^+^, 44), 277 (22), 276 (100), 275 (27), 215 (10), 77 (3). Anal. Calcd. for C_23_H_20_N_4_O_2_S (416.13): C, 66.33%; H, 4.84%; N, 13.45%; found: C, 66.12%; H, 5.03%; N, 13.62%.

*(Z)-5-((5-Methylthiophen-2-yl)methylidene)-2-morpholinothiazol-4(5H)-one* (**6d**). Orange solid (85%), m.p. 206–208 °C; FT-IR (KBr), υ: (C=O) 1673 and (C=N, C=C) 1599, 1574 cm^−^^1^; ^1^H-NMR (CDCl_3_), δ: 2.57 (s, 3H, CH_3_), 3.65 (br. s, 2H, N-CH_2_), 3.84 (br. s, 4H, –CH_2_–O–CH_2_–), 4.08 (br. s, 2H, N-CH_2_), 6.83 (d, *J* = 3.60 Hz, 1H, H-4') 7.18 (d, *J* = 3.60 Hz, 1H, H-3'), 7.91 (s, 1H, H-6) ppm; ^13^C-NMR (CDCl_3_), δ: 15.9 (–CH_3_), 48.6 (N-CH_2_), 48.8 (N-CH_2_), 66.2 (–OCH_2_–), 66.4 (–OCH_2_–), 124.6 (C-5), 125.2 (C-6), 127.1 (C-4'), 133.0 (C-3'), 137.0 (C-2'), 146.1 (C-5'), 174.8 (C-2), 180.5 (C=O) ppm. MS (IE, 70 eV) *m/z* (%): 294 (M^+^, 22), 156 (9), 155 (12), 154 (100), 153 (27), 121 (10), 97 (8). Anal. Calcd. for C_13_H_14_N_2_O_2_S_2_ (294.05): C, 53.04%; H, 4.79%; N, 9.52%; found: C, 53.21%; H, 4.93%; N, 9.46%.

### 3.3. Antifungal Activity

*Microorganisms and media*: For the antifungal evaluation, reference strains from the American Type Culture Collection (ATCC, Rockville, MD, USA), and Culture Collection of Centro de Referencia en Micología-CEREMIC (CCC, Facultad de Ciencias Bioquímicas y Farmacéuticas, Suipacha 531-(2000)-Rosario, Argentina), were used: *C. albicans* ATCC 10231, *C. tropicalis* CCC 191, *S. cerevisiae* ATCC 9763, *C. neoformans* ATCC 32264, *A. flavus* ATCC 9170, *A. fumigatus* ATTC 26934, *A. niger* ATCC 9029, *M. gypseum* CCC 115, *T. rubrum* CCC 110, *T. mentagrophytes* ATCC 9972. Strains were grown on Sabouraud-chloramphenicol agar slants at 30 °C, maintained on slopes of Sabouraud-dextrose agar (SDA, Oxoid, Hampshire, UK), and subcultured every 15 days to prevent pleomorphic transformations. Inocula were obtained according to reported procedures and adjusted to 1–5 ×10^3^ colony forming units (CFU)/mL [13,14].

### 3.4. Antifungal Susceptibility Testing

Minimum Inhibitory Concentration (MIC) of each compound was determined by using broth microdilution techniques following the guidelines of the CLSI for yeasts [13] and for filamentous fungi [14]. MIC values were determined in RPMI-1640 (Sigma, St. Louis, MO, USA) buffered to pH 7.0 with MOPS (Sigma). Microliter trays were incubated at 35 °C for yeasts and hyalohyphomycetes and at 28 °C for dermatophyte strains in a moist, dark chamber; MICs were recorded at 48 h for yeasts, and at a time according to the control fungus growth, for the rest of fungi. The susceptibilities of the standard drugs ketoconazole, terbinafine, and amphotericin B (obtained from Sigma-Aldrich, St. Louis, MO, USA) were defined as the lowest concentration of drug which resulted in total inhibition of fungal growth. For the assay, compound stock solutions were two-fold diluted with RPMI-1640 from 250 to 0.24 μg/mL (final volume = 100 μL) and a final DMSO (Sigma) concentration <1%. A volume of 100 μL of inoculum suspension was added to each well with the exception of the sterility control where sterile water was added to the well instead. MIC was defined as the minimum inhibitory concentration of the compound, which resulted in total inhibition of the fungal growth. Minimum Fungicide Concentration (MFC), the concentration of compound that kills fungi rather than inhibits the fungal growth, was determined by plating by duplicate 5 µL from each clear well of MIC determinations, onto a 150 mm SDA plate. After 48 h at 37 °C, MFCs were determined as the lowest concentration of each compound showing no growth3.5.

### 3.5. Antitumor Activity

All synthesized compounds were sent to the National Cancer Institute (NCI, Bethesda, MD, USA) to evaluate the cytotoxic activity. The process was performed in two stages. The first, consisted in evaluate the compounds at a single concentration of 1.0 μM. The second stage consisted of evaluating the compounds against 60 different cell lines (melanoma, leukemia, lung cancer, colon, brain, breast, ovary, kidney and prostate). The test consisted in a protocol of 48 h of continuous drug exposure using sulforhodamide B (SRB) protein assay to estimate cell growth [15].

## 4. Conclusions

New hetaryl- and alkylidenerhodanine derivatives **3a**–**e**, and **4a**–**d**, **5a**–**d** and **6a**–**d** were prepared from heterocyclic aldehydes **1a**–**d** or acetaldehyde **1e**. The compounds were screened by the US National Cancer Institute (NCI) to assess their antitumor activity against 60 different human cancer cell lines. Compound **3c** showed high activity against HOP-92 (Non-Small Cell Lung Cancer), which was the most sensitive cell line, with GI_50_ = 0.62 μM and LC_50_ > 100 μM from the *in vitro* assays. *In vitro* antifungal activity of these compounds was also determined against 10 fungal strains. Compound **3e** showed high activity against yeasts and dermatophyte strains, displaying the lowest MIC against *Saccharomyces cerevisiae* (MIC = 3.9 μg/mL). It is worth to take into account that we have found two interesting compounds: **3e**, that appears to be an antifungal candidate for future research, and compound **3c**, that could be an interesting molecule for the design of new hetaryl-methylidenerhodanine antitumor derivatives. Due to these significant results, we have carried out chemical studies seeking structures that enhance the antifungal and antitumor activities.
